# Protocol for developing a dashboard for interactive cohort analysis of oral health-related data

**DOI:** 10.1186/s12903-023-02895-2

**Published:** 2023-04-24

**Authors:** Vinay Sharma, Oscar Cassetti, Lewis Winning, Michael O’Sullivan, Michael Crowe

**Affiliations:** Division of Restorative Dentistry and Periodontology, Dublin Dental University Hospital, Trinity College Dublin, Dublin 2, Ireland

**Keywords:** Dashboards, R Markdown, Shiny, Data visualisation, Oral health

## Abstract

**Introduction:**

A working knowledge of data analytics is becoming increasingly important in the digital health era. Interactive dashboards are a useful, accessible format for presenting and disseminating health-related information to a wide audience. However, many oral health researchers receive minimal data visualisation and programming skills.

**Objectives:**

The objective of this protocols paper is to demonstrate the development of an analytical, interactive dashboard, using oral health-related data from multiple national cohort surveys.

**Methods:**

The flexdashboard package was used within the R Studio framework to create the structure-elements of the dashboard and interactivity was added with the Shiny package. Data sources derived from the national longitudinal study of children in Ireland and the national children’s food survey. Variables for input were selected based on their known associations with oral health. The data were aggregated using tidyverse packages such as dplyr and summarised using ggplot2 and kableExtra with specific functions created to generate bar-plots and tables.

**Results:**

The dashboard layout is structured by the YAML (YAML Ain’t Markup Language) metadata in the R Markdown document and the syntax from Flexdashboard. Survey type, wave of survey and variable selector were set as filter options. Shiny’s render functions were used to change input to automatically render code and update output. The deployed dashboard is openly accessible at https://dduh.shinyapps.io/dduh/. Examples of how to interact with the dashboard for selected oral health variables are illustrated.

**Conclusion:**

Visualisation of national child cohort data in an interactive dashboard allows viewers to dynamically explore oral health data without requiring multiple plots and tables and sharing of extensive documentation. Dashboard development requires minimal non-standard R coding and can be quickly created with open-source software.

## Background

Open data science tools and a working knowledge of data analytics is becoming increasingly important in the digital health era [1–4]. Data visualisation is a widely accepted means of deriving insights from large data-sets with complex variables [5, 6]. Dashboards have been defined as “a visual display of the most important information needed to achieve one or more objectives, consolidated and arranged on a single screen so the information can be monitored at a glance” [7]. While data-driven dashboards are commonly used as an information exploration and communication tool in a wide range of domains this tool has not been widely deployed in oral health epidemiology [2, 3, 8]. Interactive dashboards may be the ideal data technique for presenting and disseminating health-related information in an accessible format [9–11]. National cohort surveys routinely collect data from different developmental age groups to monitor and track the development and health of children. While there are obvious benefits for policy makers in analysing national survey data, there is also an onus on researchers to extract and disseminate findings in a useful and accessible manner [9, 12]. The impact of these national cohort surveys on improved understanding of the factors affecting child health and well-being outcomes has been acknowledged as they continue to inform health policies and strategies [12]. Monitoring and analysing large-scale observational data can help improve our understanding of the risk factors associated with oral disease [13–16]. Deploying multiple sources of population-level oral health data in an accessible dashboard could help monitor trends, explore risk factors and provide support for policy decisions. Extracting information from large-scale survey data-sets using summary statistics and simple contingency tables with open-source programming languages such as R is relatively straightforward and computationally reproducible [1, 17]. Ultimately, what is desirable is increased access for researchers, clinicians and policy makers to reported data analysis-visualisation that could benefit individual and population oral health [2, 18].

For an interactive analytical dashboard, the data is presented (on the same or separate screens) in a visual format that supports exploration and interrogation. The viewer can change the underlying parameters and run the document to see the output or allow for incremental updates of the dashboard as the input data changes [19]. This increases the potential impact of the data analysis by allowing interactivity and dynamic visualisations in “real-time” [6]. Communicating data analysis output through a dashboard can improve public awareness of research output and encourage greater transparency with other researchers who are able to easily assess initial findings. Integration with Git (a version control system) and deployment via GitHub pages (web pages hosted through a Github repository) promotes rapid communication, enhances collaboration and improves transparency and reproducibility. The process of creating a dashboard is described as “dashboarding” and has been outlined in detail by multiple authors [7, 8].

Flexdashboard [20] is a package developed for R Markdown which, in turn, has been described as an ecosystem for creating computational documents in R [19]. A flexdashboard layout can be created from a template within an R markdown file. This template can be customised to provide the type of framework for visual display of the elements of the dashboard which can be either dynamic or static. Adding R Shiny [21] to the flexdashboard provides reactivity which allows viewers to change the parameters or variables and immediately see the results providing a dynamic rather than static dashboard. The main focus of this protocols paper is to demonstrate the development of an analytical, interactive dashboard for researchers, policy makers or those interested in exploring national cohort surveys who may not have sufficient training in data analytics or coding skills. We demonstrate the development and deployment of the dashboard with R Markdown, Shiny and Flexdashboard, using oral health-related data from multiple Irish national child surveys.

## Methods

### Pre-design evaluation

The objective in pre-design was to decide on the structure and data requirements, user interaction process, visualisation design and key variable selection. The primary audience for the dashboard is the research community, including policy makers, who are interested in child oral health. The primary goal was to develop a dashboard to explore multiple variables associated with child oral health using the data-sets indicated in Table 1.

Simple interaction by adding an input sidebar in the input structure allows the end-user to facet the data through filters before ‘knitting’ the R Markdown document and updates visualisations automatically as the user changes inputs. Simple statistical descriptives including frequency tables and bar plots along with further displays stratified by gender and socioeconomic status were selected for input.

### Dashboard design

The R statistical programming language was used (within the R Studio integrated developer environment [22]) to import, clean, join and aggregate the data from the different data surveys detailed in Table 1. R Markdown which is a markup language, was used for basic data wrangling and statistical analysis. The flexdashboard package was used to create the structure-elements of the dashboard and the Shiny package, which allows for interactivity, was embedded within the Flexdashboard framework within an R Markdown notebook (v.1.1.456) [19, 21].

**Table 1:** Data sources

Survey	Initial sample size	Year of survey	Wave number	Child age (years)
Growing Up in Ireland infant cohort^a^	11134	1999	2, 3 and 5	3, 5 and 9
Growing Up in Ireland child cohort^a^	8500	1999	1, 2 and 3	9, 13 and 17/18
National Children Food Survey II^b^	594	2017	2	5 to 12

^a^ https://www.growingup.ie/about-growing-up-in-ireland/

^b^ https://www.iuna.net/surveyreports

Consistency in presenting data in the dashboard is important for conveying information and this includes both visual consistency of the elements and data consistency of the levels. Visual consistency was handled through Flexdashboard and using functions to generate different graphs. Each function takes a variable to display and use consistent graphics parameters. Different surveys had different data designs, instruments, data structures and taxonomy. The key variables selected were chosen based on their known associations with oral health, cariogenic food and drink and weight status [13, 23–26]. These included: dental behaviour variables such as reported frequency of tooth brushing, dental attendance and reported history of fillings and extractions; dietary intake of food and drinks typically high in free sugars and BMI status [27]. Visualisation design was simplified to standard frequency tables and bar-plots to provide easy interactivity for examining potential variable associations.

### Data sources

The data sources were static and included the national child cohort surveys listed in Table 1. The main data source was the national longitudinal survey of young people in Ireland, the Growing Up in Ireland (GUI) survey. This is a nationally representative survey which started collecting data in 2007 from a child cohort at 9 years of age. The second National Children’s Food Survey (NCFSII) was carried out by the Irish Nutrition Universities Alliance (IUNA) in 2017/18 and included details on food and nutrient intakes, body weight, physical activity and eating behaviours in children aged 5-12 years.

Full details of the surveys are available at the links provided in Table 1. The GUI and IUNA/NCFSII surveys were conducted according to guidelines laid down in the Declaration of Helsinki. Ethical approval for the GUI project was received from a Research Ethics Committee convened by the Department of Health and Children while approval for the IUNA project was obtained from the Clinical Research Ethics Committee of the Cork Teaching Hospitals, University College Cork. Data from GUI was obtained from the Irish Social Science Data Archive (ISSDA, University College Dublin, (http://www.ucd.ie/issda)) and the NCFSII data files were obtained from the IUNA Database Management Committee.

### Data preparation and workflow

**Fig. 1:**
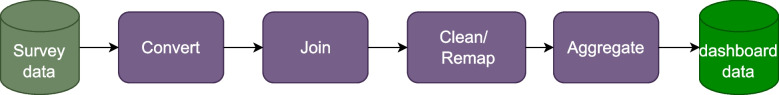
Workflow from data import to dashboard development

Data preparation and workflow is outlined in Fig. 1. All data-sets were imported from either .sav or .csv formats using R Studio [22]. All documents related to this project (except the original data files) were version controlled using GitHub (with Git installed locally) via the R Studio integrated development environment (IDE) or terminal command line. Wrangling (tidying and transformation) of the data was carried out for each individual wave before joining waves for longitudinal analysis. All data used for this analysis was unweighted. The R Markdown files and packages created for importing data files, variable renaming and renaming convention, full joins for longitudinal analysis and simple descriptive analysis including frequency tables and bar plots are available for download at https://github.com/oral-health-nutrition/oral-health-nutrition.github.io.git

Programmatic renaming and conversion from the original data frames to a tidy data frame was carried out in R Markdown to help track lineage of the transformations and integration of code with text. The data were remapped or relabelled to provide more useful descriptive names and ensure the response levels were correctly matched before the data were aggregated [17]. The main grouping variables were gender and social class. Specific functions were created to generate bar-plots and tables. The dashboard was then populated with the data and tested before publishing with version control. Only subjects who participated in all waves of the GUI cohort surveys were included.

Ontology mapping minimises differences between survey questions from different waves, or different surveys, aimed at capturing the same information [28]. While official data governance provided information about instruments used in questionnaires, ontology was not defined for many variables and the data were not harmonised between waves. For secondary analysis, the data harmonisation included remapping (reducing to common factors (using the forcats package) and custom dictionaries for programmatic mapping. Variables which were derived from different instruments such as dietary intake measurement or variables where the response levels did not match were remapped to facilitate cross wave comparisons or for longitudinal analysis. Response levels were also reordered with custom configuration for effective data visualisation. Variables not reported directly such as BMI classification were calculated. The data were aggregated mainly using tidyverse packages such as dplyr and summarised using ggplot2 and kableExtra.

## Results

### Dashboard elements

**Fig. 2:**
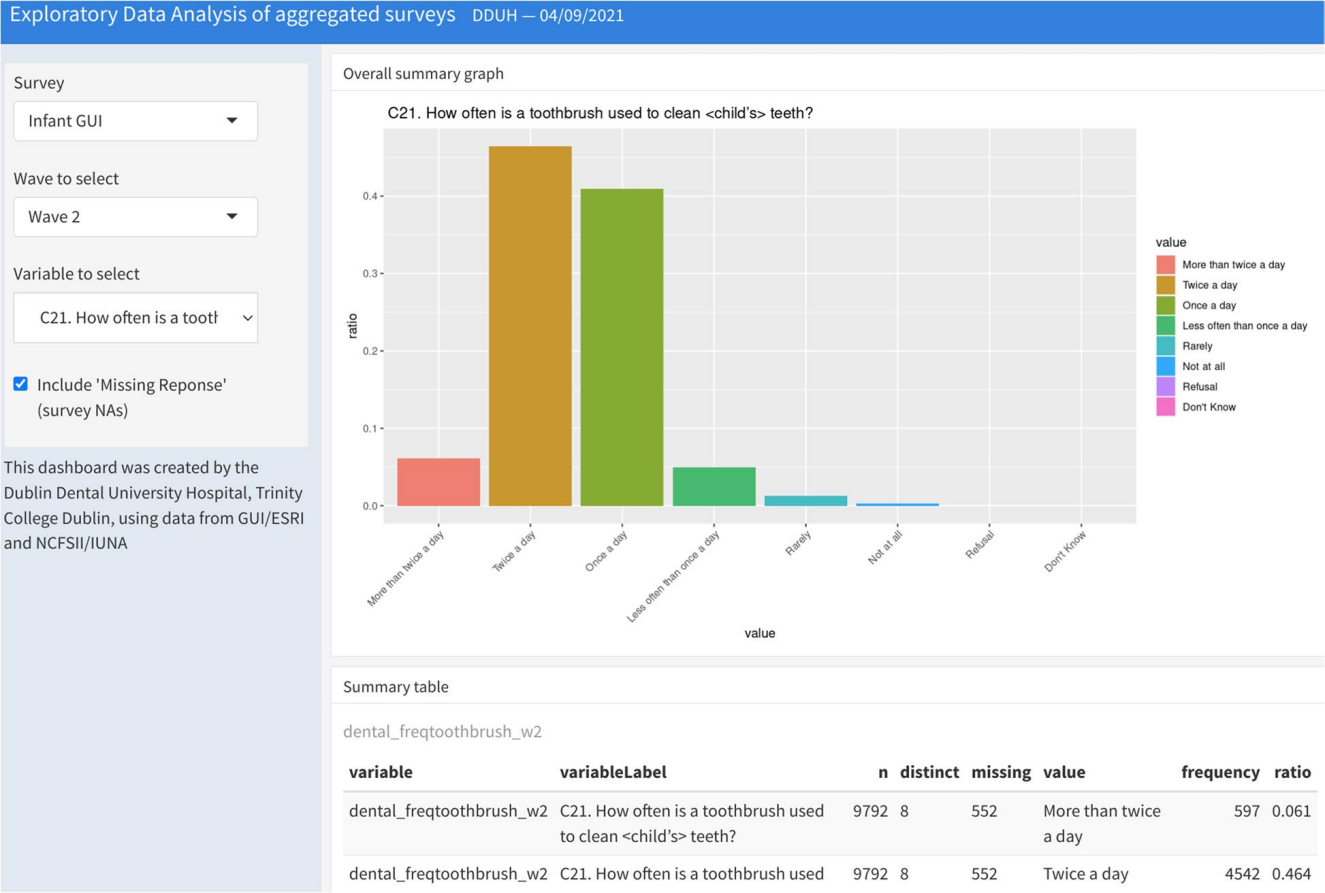
Dashboard elements

The dashboard layout is structured by the YAML (YAML Ain’t Markup Language) metadata at the start of the R Markdown document and the syntax from Flexdashboard. The YAML header controls the file format and output style. The other elements of the dashboard included input, functions and data as highlighted in Fig. 2. The aggregated data files were compiled in .RDS files which are more efficient when using R.

The Flexdashboard template (flex_dashboard) was selected within a new R Markdown document. The Shiny package was also loaded here (runtime: shiny). The general guide about the layout is that a page is generated by a first-level section while a column or row is generated by a second-level section [21]. The third-level section generates a box that contains dashboard components [19]. The layout was set to ‘vertical layout: scroll’ to specify scrolling through multiple charts and the default column orientation set to ‘orientation: rows’. Theme appearance and colours were set using the default ‘theme: cosmo’. An input sidebar was added by inserting the ‘{.sidebar}’ attribute to the first column (which also generates a typical Shiny-based dashboard). Survey type, wave of survey and variable selector were set as filter options in the drop-down column (Fig. 2). To make an output reactive, we used Shiny’s render functions which changes input to automatically render code and update output.

The final R Markdown document was rendered (using the ‘Run’ button) into a HTML document to view the dashboard locally. However, this produces a static HTML file. To create reactivity the flexdashboard must be deployed using a web server.

### Deployment of dashboard

The dashboard can be deployed using a personal or Shiny server. A Shiny account (free version) was set up at https://www.shinyapps.io/ to enable publishing using an online service for hosting Shiny apps in the cloud. The repository hosting service Github also provides free web hosting on GitHub pages (https://pages.github.com/) which is neatly integrated with R Studio and is useful for hosting documentation related to the dashboard or deploying a server-less dashboard. Essentially, GitHub Pages are public webpages hosted and published through GitHub. R Studio Connect is R Studio’s publishing platform and the R Studio IDE has push-button publishing which conveniently provides sharing of Shiny applications with R Studio Connect for R Markdown reports and dashboards. The dashboard and can be accessed at https://dduh.shinyapps.io/dduh/.

**Fig. 3:**
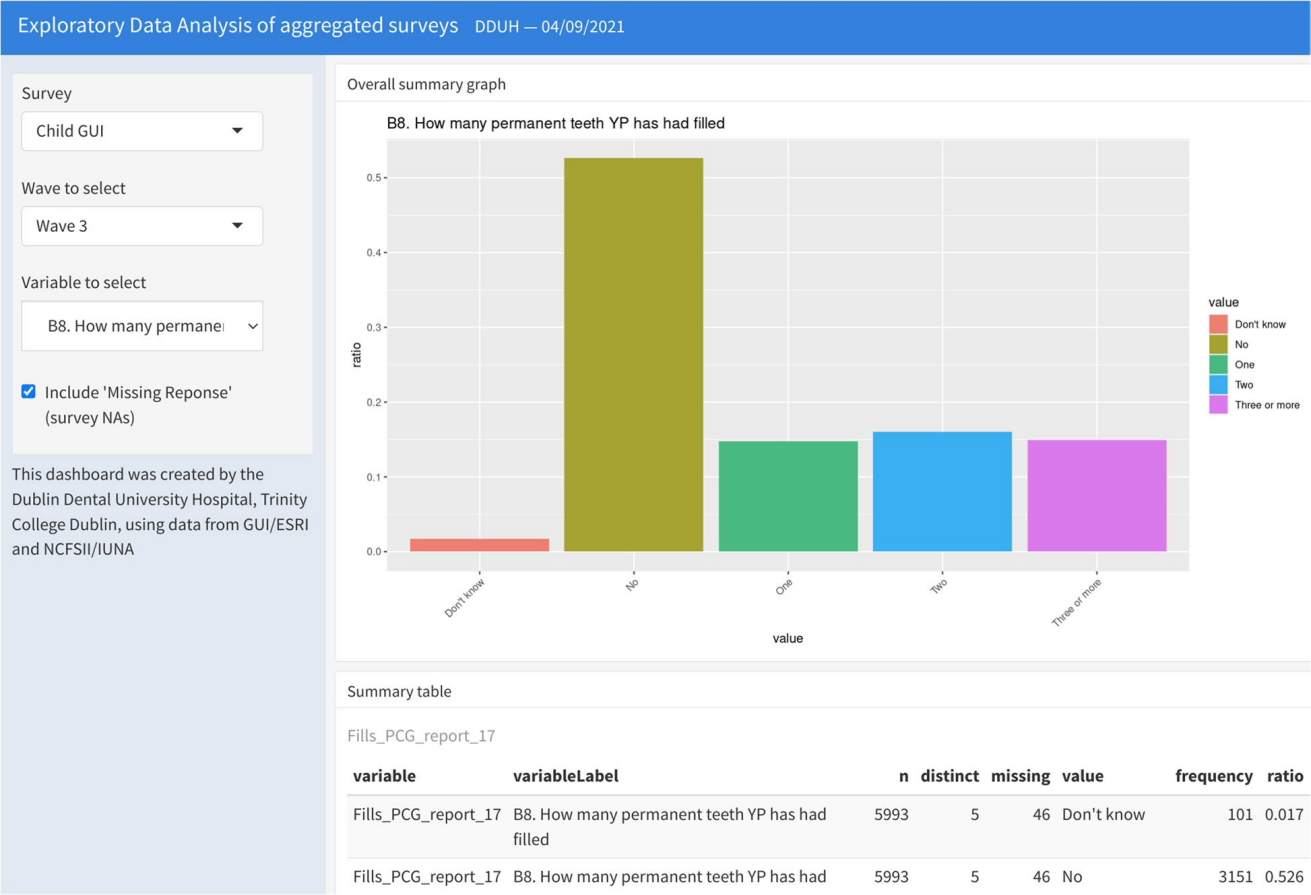
Response frequency of dental fillings in GUI child cohort 17/18 years old (wave 3)

**Fig. 4:**
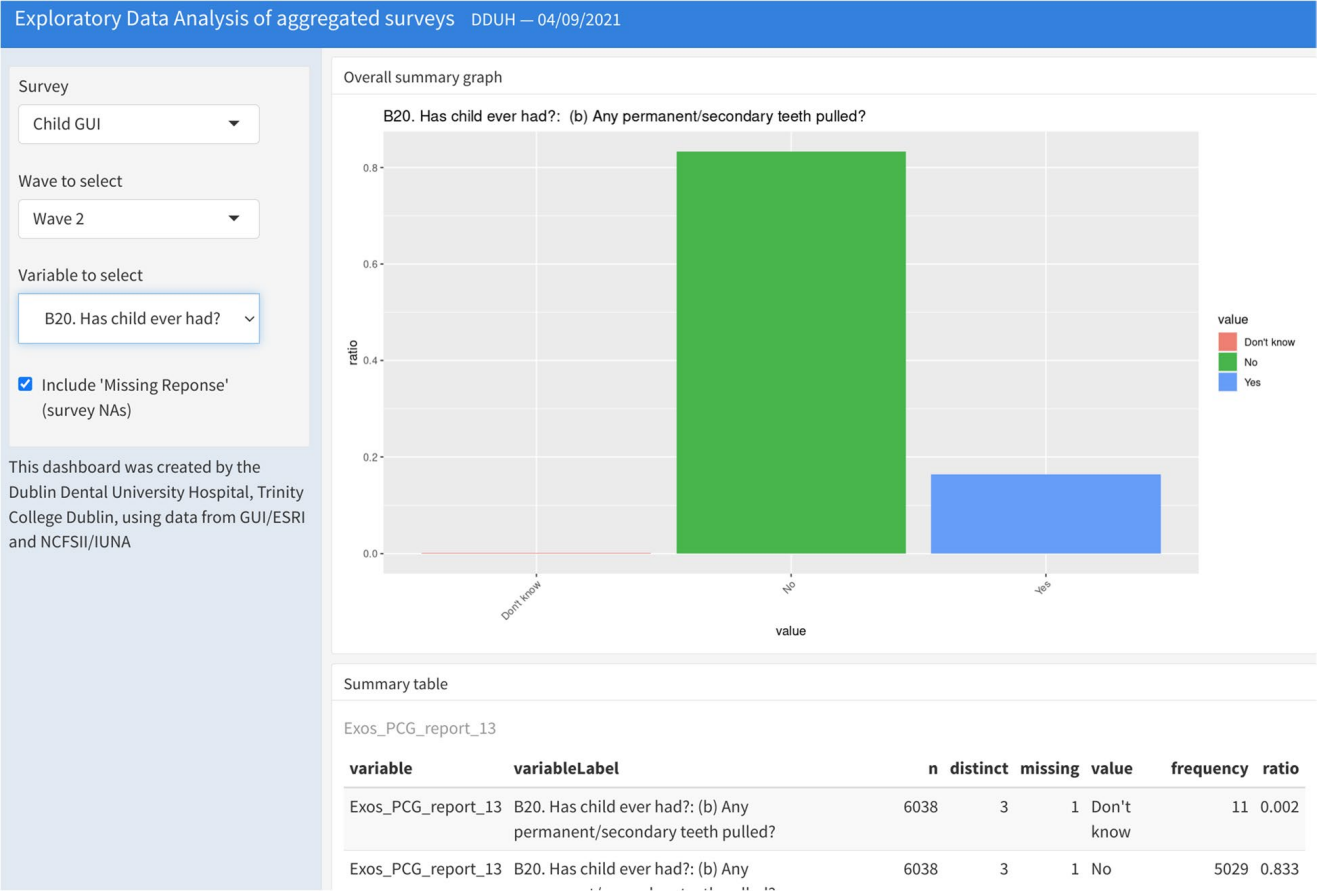
Response frequency of dental fillings in GUI child cohort at 13 years old (wave 2)

**Fig. 5:**
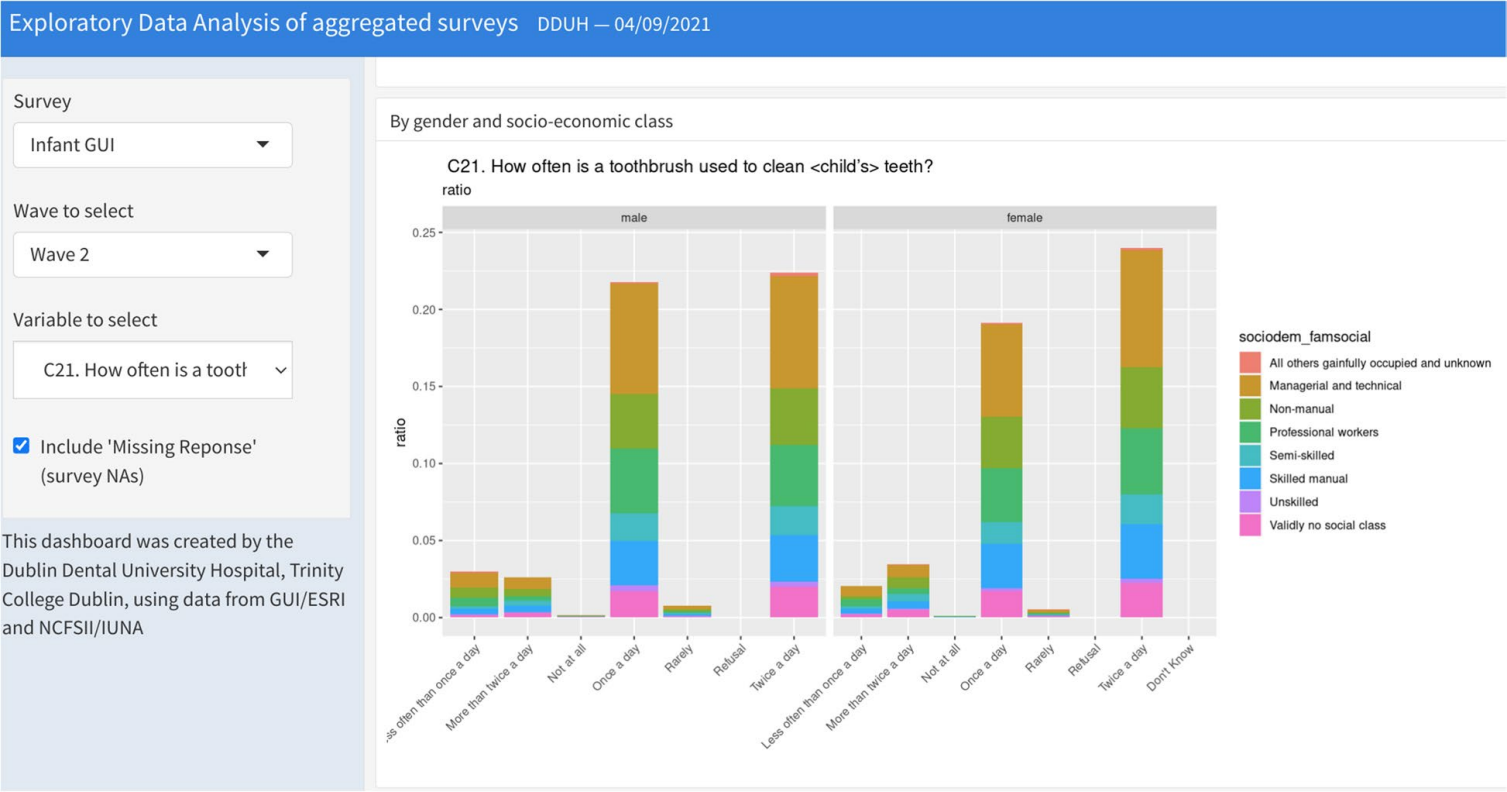
Tooth brushing frequency by gender and socio-economic status

Adding interactive controls or reactive expressions to the dashboard with Shiny generates the dynamic aspects of the components of the dashboard. Variables of interest to the viewer for a specific cohort wave (for GUI data) can be selected using the ‘survey’, ‘wave’ and ‘variable to select’ filters in the input panel. Once selected the dashboard generates output including bar-plots (and tables) for the selected variables. For example, for the GUI Child cohort at wave 3 (17/18 years old), the response is the number of fillings for the young person (Fig. 3). At wave 2 (13 years old) the response was a binary (Yes/No) if the child had dental fillings carried out (Fig. 4). For any variable of interest across any survey or wave the output is automatically stratified by gender and socio-economic status (Fig. 5).

## Discussion

This paper demonstrated the development of an interactive dashboard which provides a template for other researchers to incorporate as part of their own data visualisation and dissemination. The R Flexdashboard framework with some Shiny attributes included creates a reactive document which can be deployed with a basic knowledge of R. Visualisation of national child cohort data in dashboards allows viewers to dynamically explore oral health related variables from several Irish cohort surveys without requiring multiple plots and tables and sharing of extensive documentation.

There is little empirical data demonstrating the advantages of using data dashboards for public health policy or decision making [3, 18, 29]. However, we suggest that there are a number of advantages to this type of data visualisation including; easy and interactive communication of descriptive statistics, data-driven hypothesis generation, faster dissemination of results, computational reproducibility, incremental (addition of further surveys) and accessible across multiple devices with potential for interactive learning.

As a research tool the dashboard is a rapid means of communicating exploratory descriptive results, an overall summary of variables or data-driven hypothesis generation. Obervation of longitudinal changes within cohort survey participants and inter-survey comparisons of similar variables can provide useful insights into age or cohort changes occurring in developing children. For example, for national representative surveys, comparing the prevalence of dental fillings or frequency of tooth brushing across cohort waves is a useful descriptive measure for policy makers. Similarly, a readily accessible measure of the trends in other oral health behaviours such as dental attendance or dietary consumption of sugar-sweetened beverages can provide support for public health action.

In the R Studio environment version control is neatly integrated with Git which is hosted on GitHub. Git is a software version control system which allows researchers and collaborators to keep track of the code produced and the changes that any of the research team make to it. Version control is an essential element of any reproducible research project including R Markdown files used to create this dashboard. With a GitHub account (and Git installed) a repository can be created and linked to an R project as used in this dashboard project. As well as improving computational research reproducibility GitHub Pages can also be used to share other static content that can be ‘pushed’ from the repository such as a project related blog to improve visibility of the research and sharing of the R code used.

Another useful feature is that the dashboard can be displayed on multiple devices including tablets and mobile phones. This may provide an enhanced user experience for teaching environments for example and provide some interactive learning opportunities. It is important to note that while Flexdashboard provides an easy template to begin using dashboards there are more customisation options when using Shinydashboard alone to create a dashboard. While storing data in SQL (Structured Query Language) database would be more optimal, current restrictions on accessing the data files used in this manuscript do not permit this.

One of the issues in health sciences research is the lack of training of researchers whose primary field is neither visual design or data analytics [10, 29]. Some commentators have proposed that data literacy should be a core competence in dental curricula [2]. Data wrangling which describes the tidying and transformation of data is the most time-consuming and, arguably, demanding programming task compared to analysis and modelling [30]. Many of the toolbox of skills now required by researchers include an array of data techniques to allow them to reshape, transform, visualise and disseminate data. While a basic knowledge of R is sufficient for dashboard development the learning curve for this more complex skill-set is steep. Many of the open data science tools used in this project including distributed version control systems such as Git/GitHub, R Markdown for literate programming and RStudio Projects are regarded as essential to meet the current standards for collaborative, efficient data analysis and communication. We suggest that, given the exponential growth of digital data, dental researchers need to be prepared for a more data-driven approach whether utilising novel or more traditional surveillance data [3, 10]. However, most dental researchers face a number of obstacles when analysing and disseminating research findings from secondary data including difficulties with data sharing, user capabilities, data literacy and standardisation [2, 3].

Developing an effective dashboard is only the first stage in communicating and disseminating research findings. Strategies to improve the uptake of dashboards and training for both researchers and end-users is essential to achieve any real beneficial outcomes [29]. However, data dashboards appear to be a promising means of sharing and collaborating and are an example of how literate programming and dynamic documents can contribute to research reproducibility.

## Conclusions

Dashboard development requires minimal non-standard R coding which can be quickly created with open-source software. This manuscript provides an illustration of, and guidance for, the deployment of such a dashboard as a means of communicating descriptive results or for hypothesis generation for factors related to oral health.

## Data Availability

Data is available on application to the ISSDA - www.ucd.ie/issda. Scripts are available at https://oral-health-nutrition.github.io/bmc-2022-protocol-dashboard-gui/gen_dashboard.

## Abbreviations

IUNA: Irish Nutrition Universities Alliance
GUI: Growing Up in Ireland
NCFSII: The second National Children’s Food Survey
ISSDA: Irish Social Science Data Archive
IDE: Integrated Development Environment
